# Mad River Valley Dental Society

**Published:** 1860-08

**Authors:** 


### MAD RIVER VALLEY DENTAL SOCIETY.

THIS society met, according to adjournment, in the office of Dr. Pease, of Dayton, July 3d, 1860.

Members present:—Drs. Pease, Bradley and Jones, of Dayton; Watt and Paine, of Xenia; Blount and Ramsay, of Springfield; Rose, Lee and Palmer, of Urbana; and Clippinger, of Bellefontaine. The minutes of the previous meeting were read and approved. The Constitution was altered so as to provide for the election of a Treasurer and an Examining Committee. Dr. Ramsay was elected Treasurer, and the Examining Committee was filled by the election of Drs. Pease, Blount and Clippinger. Drs. G. F. Foote and J. T. Toland were invited to sit as Corresponding Members.

Dr. Blount, according to previous appointment, read an essay on "Fang-filling," which was ordered to be placed on file for publication. Dr. Rose, also by previous appointment, read an essay on "Fang-filling and the Treatment of Alveolar Abscess," which was disposed of in the same way.

Dr. Pease exhibited specimens of Chinese Bamboo, and described his method of using it in the treatment of nerve canals. This substance is highly elastic, not easily broken, and is capable of very minute division, while the smallest perceptible splinter seems to have strength sufficient to penetrate the canal. After some general exchange of ideas, the meeting adjourned till 8 P. M.

Evening Session.—Met at 8 P. M., and proceeded to the regular discussions.

"Suction plates" being first in order, there was a general and free interchange of sentiments, in brief, pertinent, and practical remarks. Difficult cases, and *failures* were reported without reserve, and all seemed more willing to receive than to give advice.

The next subject, "Fang-filling," was discussed at considerable length, giving evidence that the members were more interested in the preservation of the natural teeth than in the insertion of artificial ones.

The order of business was then suspended, and the Examining Committee recommended Dr. J. E. Jones, of Dayton, as a candidate for membership, who was unanimously elected.

Then, on invitation of the Dayton brethren, the society repaired to the dining room of the Franklin House, and partook of a most bounteous repast, hospitably furnished by them; and after this *special* matter, the miscellaneous business was transacted.

On motion of Dr. Rose, it was resolved to meet in Bellefontaine, on the first Thursday of October, 1860, at 2 o'clock, P. M., in the office of Dr. Clippinger. Drs. Palmer, Bradley and Watt, are the essayists for the next meeting. "Investing and soldering gold work,"—and "Sensitive Dentine," are the next subjects for discussion.

